# Non-Contact Optical Detection of Foreign Materials Adhered to Color Filter and Thin-Film Transistor

**DOI:** 10.3390/mi13010101

**Published:** 2022-01-08

**Authors:** Fu-Ming Tzu, Shih-Hsien Hsu, Jung-Shun Chen

**Affiliations:** 1 Department of Marine Engineering, National Kaohsiung University of Science and Technology, Kaohsiung 80543, Taiwan; 2 Department of Electrical Engineering, Feng Chia University, Taichung 40802, Taiwan; shihhhsu@fcuoa.fcu.edu.tw; 3 Department of Industrial Technology Education, National Kaohsiung Normal University, Kaohsiung 80201, Taiwan; jschen@nknu.edu.tw

**Keywords:** foreign material, laser sensor, area charge-coupled device, color filter, thin-film transistor

## Abstract

This paper describes the non-contact optical detection of debris material that adheres to the substrates of color filters (CFs) and thin-film transistors (TFTs) by area charge-coupled devices (CCDs) and laser sensors. One of the optical detections is a side-view illumination by an area CCD that emits a coherency light to detect debris on the CF. In contrast to the height of the debris material, the image is acquired by transforming the geometric shape from a square to a circle. As a result, the side-view illumination from the area CCD identified the height of the debris adhered to the black matrix (BM) as well as the red, green, and blue of a CF with 95, 97, 98, and 99% accuracy compared to the golden sample. The uncertainty analysis was at 5% for the BM, 3% for the red, 2% for the green, and 1% for the blue. The other optical detection, a laser optical interception with a horizontal alignment, inspected the material foreign to the TFT. At the same time, laser sensors intercepted the debris on the TFT at a voltage of 3.5 V, which the five sets of laser optics make scanning the sample. Consequently, the scanning rate reached over 98% accuracy, and the uncertainty analysis was within 5%. Thus, both non-contact optical methods can detect debris at a 50 μm height or lower. The experiment presents a successful design for the efficient prevention of a valuable component malfunction.

## 1. Introduction

A thin-film transistor liquid crystal display (TFT-LCD) comprises a sandwiched profile in which the central layer of a liquid crystal is between two substrates. The upper substrate combines the colorful layers of red, green, and blue in a color filter (CF). In contrast, the lower substrate is embedded with a microelectrode pixel with a data line and gate line across an electrical circuit, making up the thin-film transistor (TFT) [1]. Once a forward current flows through the electrode, the voltage of the pixel shifts the liquid crystal to switch the light in its optic direction. At the same time, a light penetrates the polarizer to filter out the random light and adjusts the brightness and darkness of the display.

A high-power exposure machine engraves the pattern of the CF in a gap from 100 to 300 μm with a deep ultraviolet light [2,3,4]. The array check tester inspects the electro pixel of the electric circuit in the gap 50 μm between the tester and TFT substrate [5]. The spin coater works with various photoresistors to inject the thin-film substrate into the narrow tolerance range, just several centimeters high—the chromaticity provides a vivid hue, colorful saturation, and level lightness [6,7].

The TFT-LCD requires large displays, and the uncertain issues in the factory’s products create a huge challenge. Significantly, the valuable photomask, the critical modulator, and the precious inject coater can cause inevitable scratches due to foreign materials [2].

An abnormal process results in foreign material on the glass surface of the substrate; consequently, accidents can happen that damage the component. Such accidents affect the performance of the electronic switch and chromatic variation in the valuable component simultaneously and without warning. Thus, this is concerning to the thin-film maker.

The debris is caused by the fragmented glass, whose shapes are similar to the shiny parts of the micro and colorful photoresistors. Certain components loosen their parts, which then automatically adhere to the glass substrate, and the other foreign material is from the random texture of the bunny suit. The statistics indicate that the debris consists of 70% fragmented glass, 20% metal particles, and 10% other materials [2]. Thus, this is a well-known production problem.

Various literature reviews have proposed a measurement height from a diversified field. Groot (2017) [8] explained that the vertical resolution was miscalculated in the measurement height provided by an interference microscope operating with a 100 Hz, 1000 × 1000 pixel camera by the sinusoidal waveform, converting the intensity to a heightmap. Consequently, the repeatability was 0.072 nm. Musaoglu et al. (2019) [9] employed an atomic force microscope (AFM) to measure the height of copper oxide (CuOx). As a result, the accurate measurement reached a mean grain height of 11 nm. Kaplan (2021) [10] researched the Si photodiode and material characterization of TiO_2_ and proposed a different technique to measure the height with an AFM. The result was 3 nm high, and the accuracy was significant. Kaplan also used X-ray diffraction to observe the polycrystalline structure. Thomas (2021) [11] investigated optical surface topography measurement methods by utilizing a scanning interferometer. The surface measurement based on optical imaging relied on an objective lens to collect diffracted rays. Moreover, Liu and He (2018) [12] developed a vehicle height system to use the transmitter and a receiver of laser detection to measure the vehicle’s height.

The paper utilizes non-contact optical inspection of the debris material by an area photosensor and laser optics to detect foreign materials, as compared to the literature review. As a result, our topology is quick and economical for detection and accuracy. Other methods, such as an AFM and SEM, measure the height. An AFM uses unique tiny probes to detect a particular interaction between the probe and the sample surface and utilizes a piezoelectric ceramic scanner with a three-axis displacement to scan the surface of the sample. Thus, an AFM can measure the nanometer scale even at a height of several angstroms. Moreover, an AFM uses Van der Waals forces to present the surface characteristics of a sample. Another frequent method of height measurement is a scanning electric microscope (SEM), which utilizes an electron beam as a light source and the electromagnetic field as a lens to obtain the surface morphology of an object by collecting, sorting, and analyzing information generated by the interaction of electrons and samples.

The proposed method in this paper can measure the height at the micro-scale and is suitable for the thin-film transistor of a factory. The paper presents an original method of utilizing fast and accurate detection to meet the requirements of mass production in a factory. A non-contact optical inspection detects foreign material in the leading TFT-LCD foundries. A side-view illumination engages the photosensor on the top to inspect the CF in the dark field. The other intercepted debris is detected on the TFT by laser scanning. Each topology significantly remedies the loss of valuable components.

## 2. Principle of Detected Debris on the TFT-LCD

**Figure 1:**
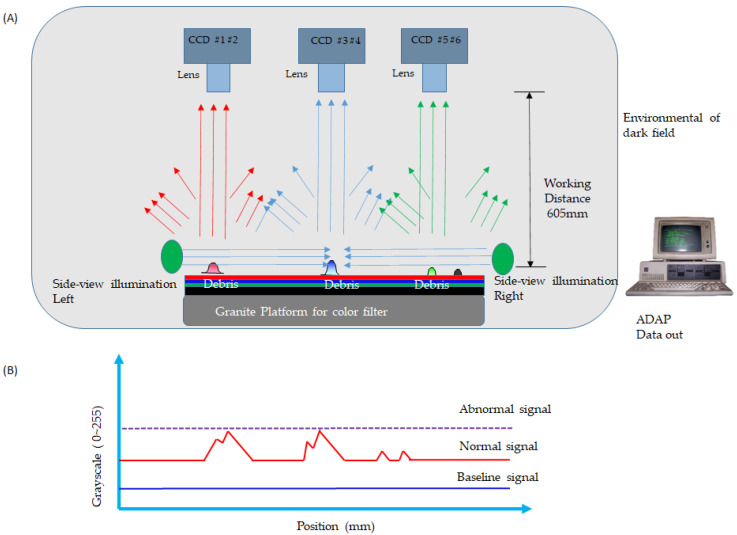
The struture of the photosensor that detects debris on the color filter (CF); (**A**) is a topology of the side-view illumination by the photosensor in the dark field, and (**B**) is the grayscale tendency.

This study utilizes a non-contact optical method to detect debris adhered to the glass substrate. The side-view illumination installed on the edges of the glass emits the opposite in the dark field. The top of the photosensor receives the reflectance shade if any suspensive debris adheres to the panel. At that moment, the mapping of the grayscale calculates the height of the protrusion on the basis of transforming the area from square to circle. Equation (1) is as follows:

(1) $$\overset{n}{\underset{i=1}{\sum }}X^{2}\times k≅\frac{\pi }{4}D^{2}$$

where *D* is the circle’s diameter/height, *X* represents the length of the effective pixel of the square based on the threshold grayscale, and *k* is a ratio value to approach both areas compared to the sample’s known height. Figure 1 illustrates the structure of the debris detection using the side-view illumination emitting a coherence light to the suspension protrusion in the dark field. Figure 1A demonstrates the area CCDs installed on the top at a specific optical work distance. The illustration shows the six sets of CCDs, #1–2, #3–4, and #5–6, indicating a field of view that covers the inspected platform depending on the inspected size of the glass generation. The side-view illumination emits non-UV light to irradiate the suspension debris. The working distance (WD) is set for the optimum resolution of the pixel size of the color filter. The granite platform is a support to prevent the deformation of the mechanism. This is a robust construction to support the glass sample. When the area CCD acquires a reflected image, the acquired dada automatic program (ADAP) can judge the protrusion and send out a warning. The protrusion found most often is debris in the CF from non-uniform photoresistors, such as the red, blue, green, and black shown in the figure.

Figure 1B demonstrates the grayscale tendency, indicating the base, normal, and abnormal signals during photosensor scanning. The abnormal signal expresses the height of the debris identified over the certainty height to compare the grayscale intensity. The topology applies to the CF; the background layer illustrates the non-transparent film. The experiment detects foreign material adhering to the substrate.

Furthermore, the surface of the glass guarantees that debris of no particular height adhere to the glass before the exposure machine; i.e., the gap is roughly 100~300 μm between the substrate and the exposure machine. When debris adheres to the CF, the ADAP detection completes the process to distribute the grayscale from 0 to 255. The granite platform maintains evenness to avoid variations due to changes in temperature. The photosensor takes the high-resolution 5 μm image (IMX204 CMOS color sensor, Sony Inc., Tokyo, Japan). The resolution is 5184 (H) × 3888 (V) at the number of recommended recording pixels. The magnification lens is 2×; thus, the optical resolution shifts to 10 μm, and, therefore, the detection is very powerful on the substrate glass. The structure detects foreign material quickly and accurately.

Moreover, the working distance (WD) expresses the height distance between the photosensor and objective for the optical resolution [12,13]. Equation (2) presents the thin-lens maker as follows:

(2) $$WD=f\times \left(\frac{M+1}{M}\right)+f\times \left(M+1\right)+(\bar{HH^{*})}$$

where *f* expresses the focal point to determine the image quality. The magnification of the image is presented by *M*; however, the coarse optical resolution is inverse to the image quality. The thickness of the optical lens in the photosensor is indicated by $\bar{HH^{*}}$. The photosensor converts the electrical signal to an image while scanning. A signal capacitor array triggers the photon electronic current in the photoactive region. The electrical current can amplify the signal to create an analog-to-digital conversion. Each capacitor accumulates electron charges in proportion to the light intensity. Alternatively, laser optics indicate foreign material adhered to the TFT in Equation (3):

(3) $$u=\{\begin{array}{c} 3.5,\;\;\;\;\;\;\;\;\;v\;\leq 3.5\\ 5.0,\;\;\;\;\;\;\;\;\;v=5.0 \end{array}.$$

**Figure 2:**
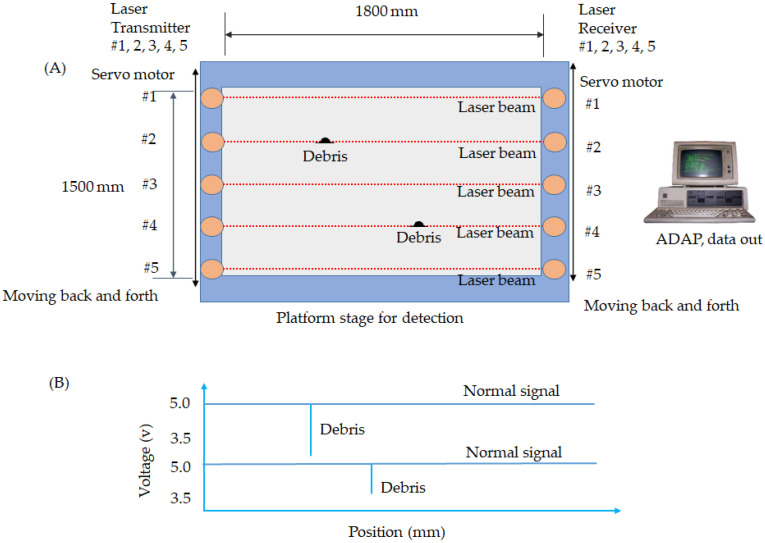
The architecture of the laser sensor illustrating the topology of a thin-film transistor (TFT), (**A**) is a topology of the foreign materials on TFT by laser optics (**B**) is a voltage tendency.

Equation (3) demonstrates a voltage variation from 5 to 3.5 V once light travel is blocked, and the debris is adhered to the substrate. The illustration of the laser in Figure 2 presents two pieces of debris adhered to the TFT substrate. The task involves five sets of laser sensors with a coherency light to detect the debris from the transmitter to the receiver in Figure 2A. The illustration demonstrates the structure of laser optics that intercepts foreign debris and is moved by a servo motor to simultaneously drive the transmitter and receiver. The red line indicates the laser’s beam, which travels to the inspected region. When the debris blocks the travel of the laser beam, the system detects the protrusion. The stage is robust enough to prevent deformation from external factors, such as temperature variations and stress and strain concentration. While the platform is moving, a sensor emits a spotlight from the transmitter. The platform is sixth-generation glass, 1800 mm × 1500 mm, which is a standard size in a TFT-LCD factory. Thus, the laser detects a sample and intercepts the debris by moving back and forth. The ADAP sends out a warning if the voltage is below 3.5 V.

In contrast to the CF, the TFT needs the laser to intercept the debris because the transparent film on the bottom layer reflects the background and interferes with the results. Thus, we chose a laser sensor to detect the debris, rather than the area CCD.

Furthermore, the light source of the laser is the red semiconductor at 670 nm (Model LX2-110W, Keyence Inc., Osaka, Japan). The transmitter emits the coherency light at 1~2.5 mm diameter of the spotlight to detect at a distance of 300~2000 mm. The response time is 0.5 ms, and the power is 12~24 VDC ± 10% ripple. Figure 2B indicates the profile of voltage variation. Once a voltage drop below 3.5 V is detected, a certain height is identified over the gap. The laser is an optical oscillator that generates light with coherence, directivity, and narrow width.

The standard deviation is known as the mean square deviation, and the mathematical symbol σ (sigma) is most commonly used in probability statistics to measure the degree of dispersion of a set of values. Thus, the higher the standard deviation, the more significant the difference among the data. This paper takes Equation (4) to analyze the difference using the ten times measurement, which quantifies the accuracy:

(4) $$\sigma =\sqrt{\frac{1}{n-1}\overset{n}{\underset{1}{\sum }}{\left(p_{i-}p\right)}^{2}}$$

where $\bar{p}$ expresses a mean value of the data set, and the symbol *n* expresses the samples. The gauge of repeatability and reproducibility (R&R) shows the measurement’s deviation [14,15,16]. The purpose of gauging R&R is to evaluate the accuracy of the measurement system and the personnel operation. Repeatability is a feature of one part measured multiple times to analyze the sum of each difference by the same operator. Reproducibility is a feature of the same part measured using measuring tools that analyze the sum of different operators [17,18]. Experimentally, the non-contact optical detection adopts the repeatability measurement [19,20].

## 3. Experimental Architecture

**Figure 3:**
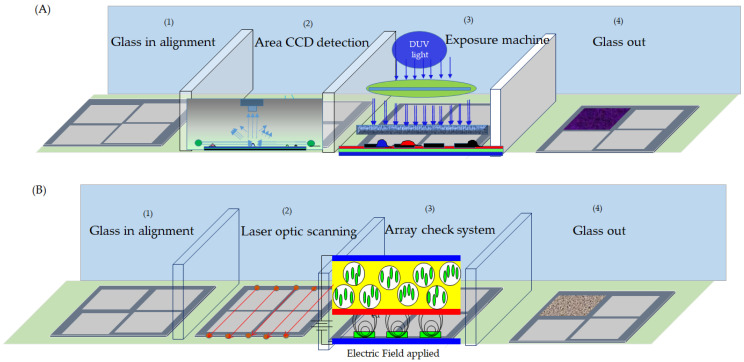
The architecture of the optical detection system comprising (**A**) of CF at (1) glass-in, (2) CCD detection, (3) exposure machine, (4) glass-out; below diagram (**B**) of TFT includes (1) glass-in, (2) laser optic scanning, (3) array check system, and (4) glass-out.

The experiment used a photo image to calculate the height of the debris on the CF and performed laser scanning to intercept foreign material on the TFT in the clean Room 10 of the factory. Figure 3A illustrates the structure for detecting the foreign material on the CF glass. First, the glass was aligned before detection in (1). The station detected the debris to calculate the height of the protrusion with a photosensor, where the photosensor was installed on the top at (2). After completing the detection, the glass was delivered to Station (3) for UV light exposure, and then the glass was delivered to Station (4). Figure 3B illustrates an interceptive topology of the laser sensor. The glass was delivered to Station (1) for alignment. Station (2) was a scanning region for the laser sensor. Station (3) performed an array check with the electrical field applied modulator. This was a non-contact detection by rotating the liquid crystal in the electronic circuit of the electrode pixel that caused the light to switch on and off. Finally, the glass was delivered out of Station (4). The experiment’s optical parameters are tabulated in Table 1. The parameter for the non-contact optical detection of the debris on the CF indicated the CCD pixel at 5 μm optical resolution applied on sixth-generation glass. The substrate size was 1850 mm × 1500 mm, which enabled the area photosensor room to cover the detectible region for six sets of CCDs. The working distance was 605 mm.

**Figure 4:**
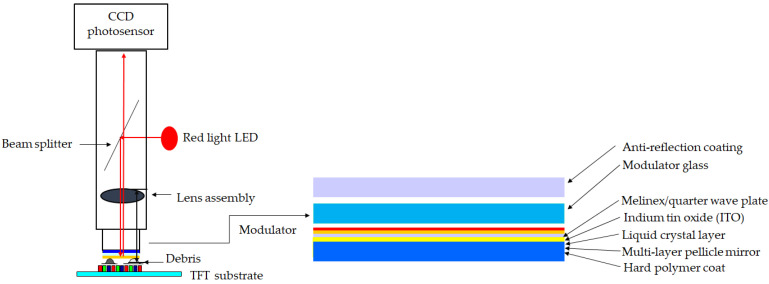
The topology of the array check system indicating the components, especially the modulator.

Figure 4 illustrates the array check system for detecting an electrode pixel in the narrow gap of 50 μm between the modulator and the substrate. Significantly, the second-to-last layer on the modulator is made of the multi-layer pellicle mirror, which is mostly a combination of zirconium (Zr) and oxygen (O). The paper will discuss the details later.

## 4. Result and Discussion

**Figure 5:**
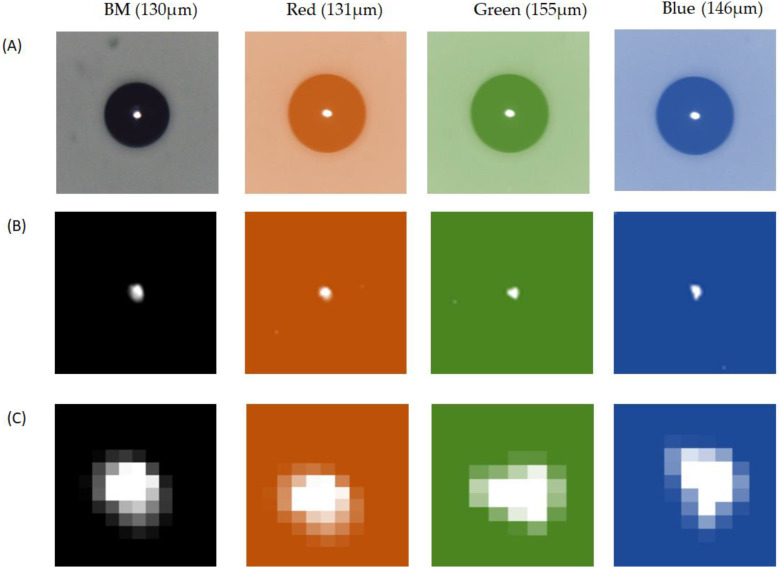
The golden sample coating the colored photoresists (PRs) with heights of 130, 131, 155, and 146 μm. Row (**A**) indicates the various solder balls magnified by microscope 10×, (**B**) indicates the grayscale image, and (**C**) is enlarged 1600% by Photoshop software.

**Figure 6:**
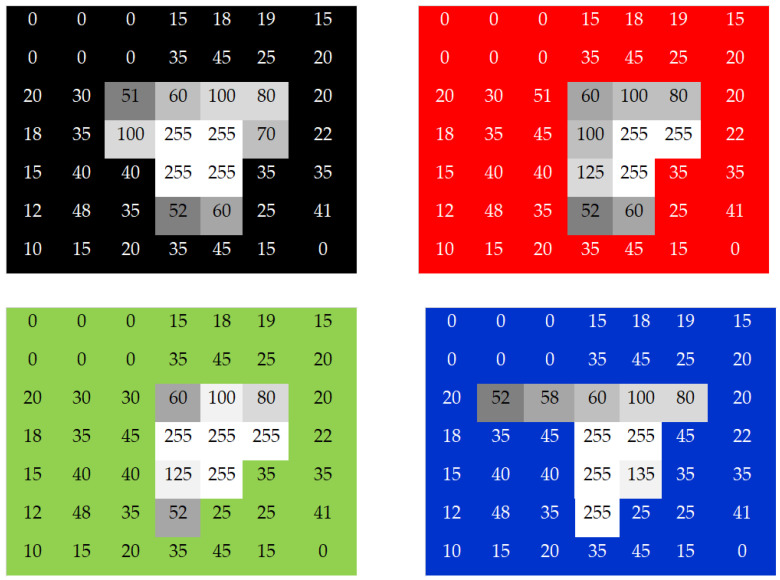
Images showing the grayscale with the threshold value for the black matrix (BM) as well as the red, green, and blue for the color filter (CF).

The experiment used the image of the CF taken by an area CCD and a laser sensor to detect the TFT debris from a factory. The task detected the height of the debris by engaging a side-view illumination to compare it with the known height from the golden sample. Figure 5 illustrates that the known height from the golden sample was 130 μm for the black matrix (BM), 131 μm for the red, 155 μm for the green, and 146 μm for the blue. Figure 5A indicates a 10× magnification of the raw image by microscope (MODEL: M-10X, Newport Inc., Irvine, CA, USA). The golden sample comprised the lead-free solder ball (S010, Unano Technology, Tainan, Taiwan). Figure 5B shows the image made by the photosensor. Figure 5C is the image enlarged 1600% by Photoshop software. Figure 6 indicates a grayscale intensity label in the color pixel of the image. As a result, the height of detection was 124 μm for the BM, 127 μm for the red, 151 μm for the green, and 145 μm for the blue. The uncertainty analysis was based on repeatability to acquire 10 times the data. Table 2 tabulates the repeatability of the BM, red, green, and blue measurements at 10 times. The measurement averages were 131, 135, 161, and 153 for the BM, red, green, and blue, respectively. After comparing the known heights from the golden sample, the result showed error levels of 5% for the BM, 3% for the red, 2% for the green, and 1% for the blue.

Since the BM is a black layer that absorbs reflective light from the environment, the PR was shown as the weakest response. Thus, the error was higher than those for the colored PRs. The most common standard deviation was applied to the probability statistics to measure the degree of statistical dispersion. Statistically, the 68–95–99.7 rule was within the normal distribution [21]. The mean percentage concerned one standard deviation, two standard deviations, and three standard deviations. At the same time, the experiment took the three standard deviations (3σ) at a 99.7% confidence level [22,23].

Furthermore, the blue PR indicated that the minor deviation in the measurement was due to short wavelength with a significant reflective response. Consequently, the 3σ indicated this at 5% for the BM, 3% for the red, 2% for the green, and 1% for the blue.

**Figure 7:**
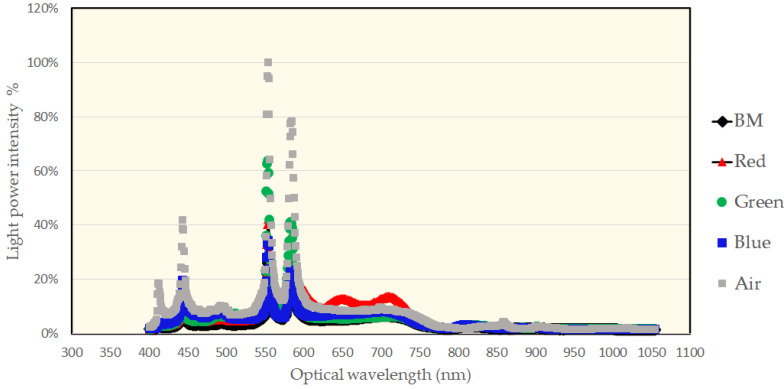
The distribution of the spectrum measures of the photoresists for the black matrix (BM), red, green, and blue.

The task filtered out UV wavelengths smaller than 400 nm to avoid overexposure of the thin film and to prevent the characteristic PR variance [24], as seen in Figure 7. The wavelength distribution illustrates the profiles for the BM, red, green, and blue. In addition, the air was a reference spectrum against which each PR response was compared. The BM demonstrated the weakest light response because of the black body. The red PR presented 40% intensity. The green PR demonstrated the highest intensity at over 60% response intensity because the green wavelength had the most vision sensitivity among the colors. The response of the blue light was around 40%.

**Figure 8:**
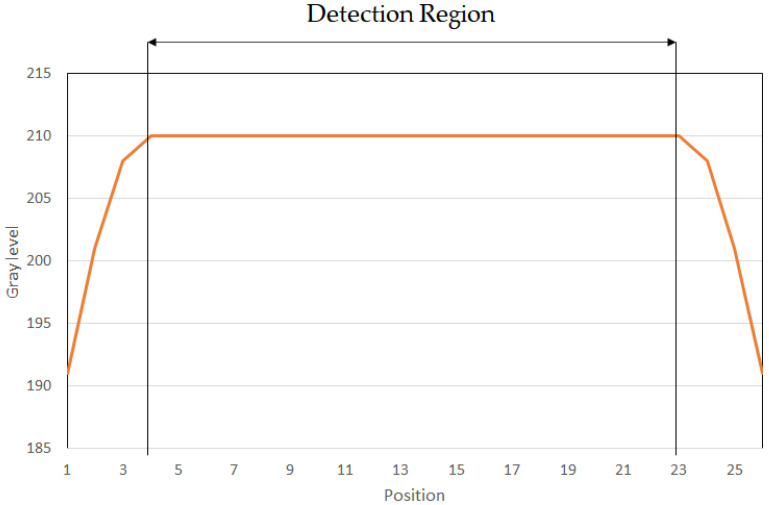
The uniformity of the side-view illumination indicating the distribution.

Figure 8 illustrates the uniform light emitted from the region that connects the multiple fibers to the light source. The start-and-end position tended toward a sloop shape; thus, we chose the uniformity brightness as a detection region.

**Figure 9:**
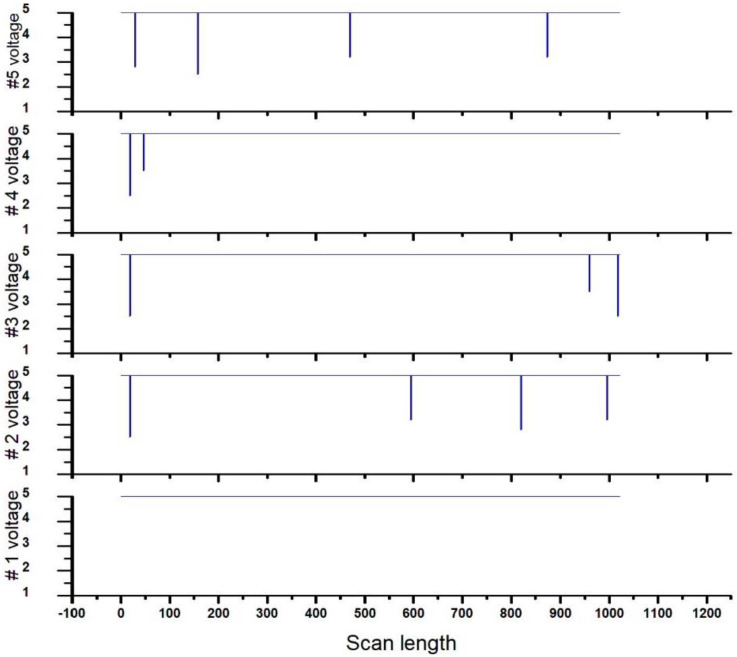
The profile of the laser sensor indicating the five sets of receivers.

The experiment used the laser sensor to scan the whole region. Figure 9 shows the laser’s tendency to intercept the debris. The horizontal lines express the scanning length using five sets of coherence light-emitting sensors across the receiver. We specifically installed a granite platform with optimized flatness to avoid the warpage. The vertical lines indicate voltage variation. Once the voltage was below 3.5 V, it was considered a successful interception on the glass of the substrate.

Moreover, the laser beam was a coherence light that traveled the detected region from transmitter to receiver above the 100 μm height of the substrate. It was an excellent choice to apply the transparent and non-transparent thin films. Otherwise, we had not used the laser optics, the reflective image with a sub-layer pattern of the TFT would have interfered with detection. Conversely, we developed the optical detection to apply laser optics to the thin-film transistors.

Moreover, the voltage for sensor #1 indicated the normal condition at 5 V. Sensors #2–5 intercepted the debris at 4, 3, 2, and 4. Accordingly, the voltage was below 3.5 V. The experiment was undertaken ten times for accuracy of the statistics. The measurements are tabulated in Table 3. The height of the golden sample is in the table. Consequently, the maximum 5% for the 3σ was a 99.7% confidence interval.

Furthermore, the modulator of the array check system was a critical component for the liquid crystal layer to inspect the electrode pixel at the dimensions of 143 mm × 133 mm. An energy-dispersive X-ray spectroscopy (EDX) was used to detect the elemental analysis and chemical characterization. The experiment was conducted with a field emission scanning electron microscope (FESEM) manufactured by PhotoMetrics, Inc. (Huntington Beach, CA, USA), which analyzed the spectrum for the modulator. Since elements emit different emission spectra because of their different atomic structures, the components were distinguished by their X-ray spectra. As a result, the most significant elements on the layer of the modulator were oxygen (O) and zirconium (Zr), i.e., O35.51Zr32.64B14.42 Si8.38C9.05 (wt.%), as investigated by EDX.

**Figure 10:**
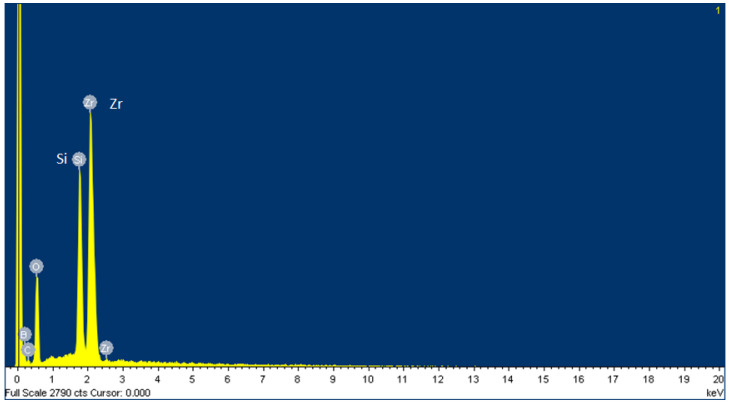
The EDX spectrum of the FISEM indicating the response of the material.

Zr is a metal element used mainly for heat-resistance and as a sunscreen agent, while a small amount of zirconium is an alloying agent to resist corrosion. Zr is the prevalent metal in electronic instruments. A Zr foil was applied in a thin film, including chemical vapor deposition (CVD) and physical vapor deposition (PVD). The thickness of the foil was approximately 2 mm on the modulator. A hard polymer coat was the bottom layer, and a multi-layer pellicle of the Zr was the second-to-last layer. Figure 10 shows the EDX spectrum, and Table 4 tabulates the presence of the constituent elements statistically.

**Figure 11:**
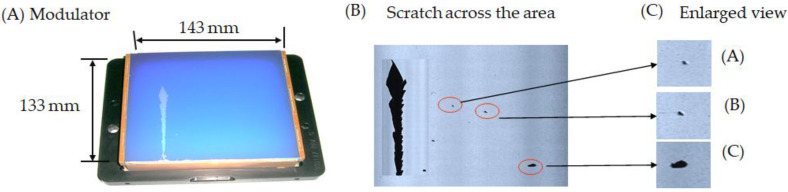
Analysis of the debris adhering to the modulator indicates (**A**) of the damaged modulator, (**B**) of scratch across the area, and (**C**) of the enlarged view.

In particular, the experiment investigated the damaged condition of the array check system modulator with a photosensor. Figure 11 illustrates the (A) typical structure of the modulator, (B) indicates a scratch across the area and several debris pieces adhered to the surface, and (C) is an enlarged view.

**Figure 12:**
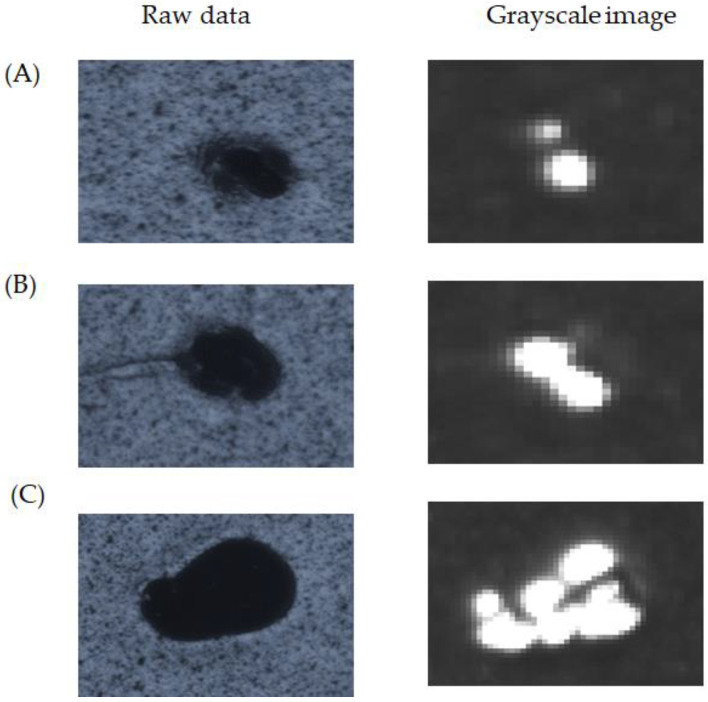
Images of the debris enlarged by the microscope indicate the known heights (**A**) 125 μm, (**B**) 250 μm, and (**C**) 700 μm.

Figure 12 shows enlarged views of the raw and grayscale images to investigate the debris on the modulator. The images underwent a magnification of 400% to show the grayscale distribution. As a result, the calculated heights (A) 123 μm, (B) 245 μm, and (C) 687 μm were the bases of a threshold of 50 for the golden sample by transforming the square to the circle. Table 5 indicates the known heights (A) 125 μm, (B) 250 μm, and (C) 700 μm. Thus, the calculated height exceeded the known height by an error of 2% with the 3σ of 2% for the repeatability measurement. Thus, the non-contact optical detection provided high accuracy, and the damage-free significance was very impressive.

## 5. Conclusions

This study experimentally investigated the non-contact optical method for detecting the height of debris on CF glass by engaging a side-view illumination using the area CCD and a laser sensor emitting a coherency light to scan the TFT. The result showed the CF accuracy was at 5% for the BM, 3% for red, 2% for green, and 1% for blue. In addition, the repeatability of the three standard deviations was at 5% for the BM, 3% for the red, 2% for the green, and 1% for the blue. Alternatively, the laser intercepting the certain height of debris adhered to the TFT was indicated at a voltage below 3.5 V. The accuracy of the repeatability was also within 5%.

Moreover, the calculated debris height indicated a 2% error over the known height and a 2% repeatability at the three standard deviations on the modulator. Thus, an optical inspection can ensure the damage-free condition of the critical component. In other words, the non-contact optical inspection extends the necessary applications.

## Figures and Tables

**Table 1:** The parameters for optical detection of the debris on the color filter.

Description	Value	Detailed	Length	Width
CCD Pixel Size (μm)	5	Substrate dimension (mm)	1850	1500
Magnification	2	Signal CCD FOV (mm)	1037	778
Optical resolution (μm)	10	Require CCD quantity	3	2
Focused lens (mm)	50	Signal overlap (mm)	252	14
Working distance (mm)	605	All overlap (mm)	533	750

**Table 2:** The height measurement as indicated ten times compared to the golden sample, including uncertainty analysis.

Glass	1	2	3	4	5	6	7	8	9	10	Avg	Calculated (μm)	Known (μm)	Error	3σ
BM	125	132	130	132	130	132	133	130	132	133	131	124	130	5%	5%
Red	135	135	132	134	135	136	135	135	136	135	135	127	131	3%	3%
Green	160	160	162	163	160	160	162	160	160	160	161	152	155	2%	2%
Blue	153	153	155	153	153	153	153	153	153	154	153	145	146	1%	1%

**Table 3:** Statistics of the uncertainty indicating the ten measurements for three standard deviations (3σ).

Laser #	1	2	3	4	5	6	7	8	9	10	Average	Height (um)	3σ (%)
1	4.99	4.99	4.95	4.99	4.95	4.99	4.95	4.90	4.98	4.98	4.97	100	2%
2	2.55	2.45	2.45	2.52	2.55	2.44	2.55	2.50	2.45	2.45	2.49	200	5%
2	3.23	3.25	3.15	3.25	3.24	3.33	3.22	3.28	3.26	3.25	3.25	200	4%
2	2.81	2.85	2.88	2.85	2.75	2.81	2.80	2.80	2.85	2.75	2.82	200	4%
2	3.22	3.15	3.23	3.25	3.15	3.15	3.25	3.21	3.10	3.15	3.19	200	5%
3	2.54	2.55	2.56	2.45	2.47	2.48	2.48	2.49	2.45	2.45	2.49	250	5%
3	3.48	3.52	3.55	3.45	3.45	3.56	3.47	3.41	3.42	3.55	3.49	250	5%
3	2.51	2.52	2.52	2.55	2.53	2.55	2.55	2.45	2.52	2.51	2.52	250	3%
4	2.45	2.45	2.55	2.45	2.55	2.45	2.45	2.50	2.45	2.44	2.47	260	5%
4	3.52	3.45	3.55	3.45	3.55	3.42	3.52	3.41	3.46	3.55	3.49	260	5%
5	2.82	2.78	2.77	2.81	2.88	2.85	2.85	2.82	2.88	2.88	2.83	280	4%
5	2.52	2.55	2.45	2.45	2.52	2.51	2.55	2.52	2.47	2.48	2.50	280	4%
5	3.25	3.22	3.32	3.27	3.28	3.25	3.26	3.25	3.22	3.21	3.25	280	3%
5	3.26	3.25	3.15	3.25	3.22	3.24	3.25	3.21	3.29	3.28	3.24	280	3%

**Table 4:** Statistics of the element combinations tabulating the weight %.

Element	O	Zr	B	Si	C	Total
**Mean**	35.51	32.64	14.42	8.38	9.05	100
**Max.**	35.51	32.64	14.42	8.38	9.05	100
**Min.**	35.51	32.64	14.42	8.38	9.05	100
**Std. deviation**	0	0	0	0	0	0

**Table 5:** Measurements of the debris on the modulator.

Debris	1	2	3	4	5	6	7	8	9	10	Avg	Calculated (um)	Known (um)	Error	3σ
A	132	130	130	130	130	129	130	128	130	129	130	123	125	2%	2%
B	260	259	260	258	260	260	258	260	256	260	259	245	250	2%	2%
C	725	730	730	735	730	730	720	725	725	730	728	687	700	2%	2%

## Data Availability

The data presented in this study are available on request from the corresponding author.
